# Genomic selection for genotype performance and environmental stability in *Coffea canephora*

**DOI:** 10.1093/g3journal/jkad062

**Published:** 2023-03-22

**Authors:** Paul Adunola, Maria Amélia G Ferrão, Romário G Ferrão, Aymbire F A da Fonseca, Paulo S Volpi, Marcone Comério, Abraão C Verdin Filho, Patricio R Munoz, Luís Felipe V Ferrão

**Affiliations:** Blueberry Breeding and Genomics Lab, Horticultural Sciences Department, University of Florida, Gainesville, FL 32611, USA; Instituto Capixaba de Pesquisa, Assistência Técnica e Extensão Rural—Incaper, Vitoria, ES 29052-010, Brazil; Empresa Brasileira de Pesquisa Agropecuária—Embrapa Café, Brasília, DF 707770-901, Brazil; Instituto Capixaba de Pesquisa, Assistência Técnica e Extensão Rural—Incaper, Vitoria, ES 29052-010, Brazil; Multivix group, Vitoria, ES 29075-080, Brazil; Instituto Capixaba de Pesquisa, Assistência Técnica e Extensão Rural—Incaper, Vitoria, ES 29052-010, Brazil; Empresa Brasileira de Pesquisa Agropecuária—Embrapa Café, Brasília, DF 707770-901, Brazil; Instituto Capixaba de Pesquisa, Assistência Técnica e Extensão Rural—Incaper, Vitoria, ES 29052-010, Brazil; Instituto Capixaba de Pesquisa, Assistência Técnica e Extensão Rural—Incaper, Vitoria, ES 29052-010, Brazil; Instituto Capixaba de Pesquisa, Assistência Técnica e Extensão Rural—Incaper, Vitoria, ES 29052-010, Brazil; Blueberry Breeding and Genomics Lab, Horticultural Sciences Department, University of Florida, Gainesville, FL 32611, USA; Blueberry Breeding and Genomics Lab, Horticultural Sciences Department, University of Florida, Gainesville, FL 32611, USA

**Keywords:** genomic selection, coffee, plant breeding, genotype-by-sequencing, genotype-by-environment, genomic prediction, GenPred, shared data resource

## Abstract

Coffee is one of the most important beverages and trade products in the world. Among the multiple research initiatives focused on coffee sustainability, plant breeding provides the best means to increase phenotypic performance and release cultivars that could meet market demands. Since coffee is well adapted to a diversity of tropical environments, an important question for those confronting the problem of evaluating phenotypic performance is the relevance of genotype-by-environment interaction. As a perennial crop with a long juvenile phase, coffee is subjected to significant temporal and spatial variations. Such facts not only hinder the selection of promising materials but also cause a majority of complaints among growers. In this study, we hypothesized that trait stability in coffee is genetically controlled and therefore is predictable using molecular information. To test it, we used genome-based methods to predict stability metrics computed with the primary goal of selecting coffee genotypes that combine high phenotypic performance and stability for target environments. Using 2 populations of *Coffea canephora*, evaluated across multiple years and locations, our contribution is 3-fold: (1) first, we demonstrated that the number of harvest evaluations may be reduced leading to accelerated implementation of molecular breeding; (2) we showed that stability metrics are predictable; and finally, (3) both stable and high-performance genotypes can be simultaneously predicted and selected. While this research was carried out on representative environments for coffee production with substantial crossover in genotypic ranking, we anticipate that genomic prediction can be an efficient tool to select coffee genotypes that combine high performance and stability across years and the target locations here evaluated.

## Introduction

Coffee is one of the most consumed beverages and traded agricultural commodities in the world. Globally, an estimated 125 million people along the supply chain depend on coffee for their livelihood (Fairtrade 2022), thereby providing 2.2 billion cups of coffee daily for its consumers. Despite this importance, in recent decades most producer countries are experiencing stagnation or even declines in production except in Brazil and Uganda where modestly higher gains have been recorded (International Coffee Organization 2020; Foreign Agricultural Service/United States Department of Agriculture 2022). This has been associated with different factors, including the lack of climate resilience, price volatility, shortage of labor resources, and susceptibility to insect infestations and diseases (World Coffee Research 2019).

Contrasting other initiatives to confront these issues, modern plant breeding techniques can meet these challenges. Conventionally, coffee breeders have centered their decisions on visual selection collected over multiple generations, which is time-consuming and costly (Melese and Kolech 2021). Modern breeding programs are transitioning to genomic-assisted breeding, with the potential of maximizing genetic gains by shortening cycles and increasing selection accuracy (Crossa *et al*. 2021). Namely, genomic selection is a form of marker-assisted selection in which molecular markers covering the entire genome are used to predict the genetic merits of individuals for quantitative traits (Atefi *et al*. 2016). In coffee, for example, genomic selection has been assessed for a restricted set of traits, showing the potential to be incorporated into the pace and scope of breeding programs (Ferrão *et al*. 2017; Ferrão *et al*. 2018; Alkimim *et al.* 2020; Fanelli Carvalho *et al.* 2020).

Jointly with genomic prediction implementation, the relevance of genotype-by-environment interaction (GEI) is an important variable when evaluating the phenotypic performance of coffee plants. For most perennial crops, genomic selection and GEI have been simultaneously evaluated to predict the phenotypic performance of target genotypes in different environments (Mageto *et al*. 2020; Fois *et al*. 2021). This is usually carried out in multienvironment trials, in which GEI can be framed in different ways. At the statistical level, methods based on ANOVA, mixed linear models, and linear-bilinear models are the most common approaches used for quantifying GEI effects (van Eeuwijk *et al*. 2016). When quantified, the magnitude of the interaction is commonly communicated in terms of trait stability (Piepho 1998), in which a genotype can be static (lacks response to change in environmental conditions) or dynamic (relative change parallel to average performance with change in environmental conditions) (Mohammadi *et al*. 2012).

Advances in analytical methods have provided the research community with a breadth of tools to quantify the relevance of GEI in plant breeding. One avenue to study GEI is to search for individuals with stable fitness across environments. To this end, the use of linear regressions, in the form of Finlay–Wilkinson (FW) regressions, has gained popularity, given its parsimonious input requirements. In FW regression, the expected performance of a genotype varies as a function of the environmental effects, and plants are either classified on their stability or responsiveness to environment potential (Finlay and Wilkinson 1963; van Eeuwijk 1995). Using a similar approach, but on a multidimensional scale, a second standard approach expresses the GEI effects using bilinear models, in the form of additive main effect and multiplicative interaction model (AMMI). A desirable property of AMMI models is that the genotypic and environmental scores can be inspected using biplot graphics that ultimately facilitate the exploration of relationships between genotypes and environments (Gauch *et al*. 2008; Oliveira *et al*. 2014).

In coffee, environmental stability is mainly addressed in terms of spatial and temporal variations (Lammerts van Bueren *et al*. 2018; Bertrand *et al*. 2021). Stable fitness across sites not only represents more uniformity between growers but can also aid in unifying the management of the crop by public breeding programs. Furthermore, growers are looking for cultivars that are less susceptible to temporal variations, one of their major complaints. Coffee usually exhibits fluctuations over production years due to biannual phenological development, a fact that can hamper selection (Monselise and Goldschmidt 1982; Rakocevic *et al*. 2018). To circumvent such issues, most coffee breeders have guided their decisions based on phenotypic measurements across multiple locations, with a minimum number of 4 harvests. However, in the context of molecular breeding implementation, both understanding the minimal number of harvests, and the potential of genome-base methods to predict trait stability, in coffee is still unknown.

Considering the importance of GEI for coffee breeding, in this study, we address the following main question: can we predict trait stability using a genome-based approach? Motivated by the potential to use genomic prediction to reshape traditional coffee breeding schemes, we used a historical data set collected in 2 breeding populations of *Coffea canephora*, evaluated in multiple years and locations, to address the following specific considerations: (1) the minimal number of harvests necessary to predict different coffee traits in a context of genomic prediction; (2) the most stable genotypes predicted across locations and years, using FW and AMMI approaches; and, (3) the best strategy to predict stability metrics when data from target populations are collected. Altogether, we hypothesized that genomic prediction could be an efficient tool to select coffee genotypes that combine high phenotypic and stable performance across years and locations.

## Material and methods

### Plant material and trait measurement

The populations used in this study were generated as part of the coffee breeding program at the Instituto Capixaba de Pesquisa, Assistência Técnica e Extensão Rural (Incaper); Espirito Santo State, Brazil; in partnership with Embrapa Café, Brazil. Two recurrent selection populations of *C. canephora* were evaluated, with different fruit maturation times, referred to here as *Premature* and *Intermediate* populations. For the *Premature* population, a total of 9 parents were used, with an average earlier fruit maturation in February to March and harvest in May. The *Intermediate* population was designed from 16 parents from the Incaper Germplasm Bank, with fruit ripening in March to April and harvest in June. Parents from both populations were established in separated and isolated seed orchards. After 1 cycle of recombination, seeds were derived from each maternal plant and planted in 2 separate fields. Progenies in both populations were first submitted to a visual screening based on yield, disease resistance, and drought tolerance. After screening, the 103 best progenies in the *Premature* population, and the 118 best progenies in the *Intermediate* population, were cloned into separate trials established in a randomized complete block design with three replications and five plants per plot (Supplementary Fig. 1). The *Premature* and *Intermediate* populations were evaluated for four consecutive harvest-production years in two environments: Marilandia Experimental Farm (FEM)—latitude 19°24 south, longitude 40°31 west, 70 m altitude and Sooretama Experimental Farm (FES)—latitude 15°47 south, longitude 43°18 west, 40 m altitude. Additional historical data was included for the FEM location, in which traits were evaluated over eight harvests. More details about the plant material and experimental design are described in Ferrão *et al*. (2017, 2018) and the Supplementary Material.

### Trait measurements

For the *Premature* and *Intermediate* populations, a total of 20 phenotypic traits were evaluated. Herein, we classified the traits into 4 categories:

#### Morphoagronomic traits

A group of 6 traits was visually scored for morphological and physiological traits related to coffee growing. The maturation time (FL) was evaluated using a 1- to 7- rating scale where 1 indicated precocity and 7 represents late maturation. Uniformity of the maturation period (UNIF) was measured on a 1 to 3 scale, where 1 represented uniformity of fruit maturation and 3 lack of uniformity. Bean size (GSIZ) was on a 1 to 7 scale, with the lowest value indicating the smallest beans. Plant architecture (PRT) was related to the size of the bush, and it was assessed using a 1 to 3 scale, where 1 meant small plant size and 3 large. Vigor (VIGOR) was a metric associated with healthiness and was evaluated using a 1 to 9 scale, where 1 indicated low vigor and 9 high vigor. Finally, the general scale (GSCE) was a visual metric assessed by breeders and indicated the overall performance of a genotype classified by breeders. It was evaluated using a 1 to 9 scale, where 1 represented poor genotypes and 9 more promising ones.

#### Disease resistance

A total of three traits were visually evaluated for disease resistance, all using a 1 to 9 scale, where 1 represents no visual symptoms and 9 visible and severe symptoms. Coffee leaf rust (RUST) is caused by the fungus *Hemileia vastatrix*, whose symptoms include pale yellow spots on upper leaf surfaces, followed by powdery orange-yellow lesions. Coffee leaf mine (LMINER) is caused by *Leucoptera coffeella*, a moth in the family *Lyonetiidae*. Cercospora leaf spot (CERC), also called Brown eye spot or Berry blotch, is caused by the fungus *Cercospora coffeicola*, and the main symptoms are brown spots on foliage, which enlarge and develop a gray-white center and red-brown margin.

#### Yield

The total production (kilograms of mature coffee fruit in the cherry stage) of each progeny were harvested and weighed. In accordance with internationally accepted practices in coffee research and production, yield was reported as bags of 60 kilograms per hectare.

#### Post-harvest

After being harvested, a sample of 2 kg of ripened fruits were processed in 2 stages. The first was to use a natural drying method (sun drying), and obtain the weight (in grams) of coffee fruits afterward, named as CHERRY_g. The second was depulping (getting rid of the skin and mucilage) and weighing the resulting dry processed and unroasted beans, herein GREEN_g. The ratio between CHERRY_g and GREEN_g was measured as a new trait, named as CHE:GREE. Because it is known that coffee bean size can affect the roasting process and influence drink quality (Seninde and Chambers 2020), for additional postharvest evaluations, we sampled 300 grams of raw beans and classified them using a set of sieves. Namely, we used a round sieve M17 (flat and large beans), round sieve M15 (flat and medium beans), round sieve M13 (medium “moca” beans), and oblong sieve M10 (small “moca” beans). We also included intermediate sieves called M11, M12, and M13. The residual (RES) measures impurities aspects presented along the sieve analyses (sticks, stones, broken grains, among others).

### Genotypic data

Genotyping by sequencing (GBS) was used to genotype the populations in the Genomic Diversity Facility at Cornell University. Leaf samples from each genotype were collected and lyophilized. DNA extraction was made using Qiagen DNeasy Plant, and the genomic libraries were prepared following Elshire *et al*. (2011). *Ape*KI restriction enzyme was used for the digestion of the DNA samples, and 96 samples were multiplexed per Illumina flow cell for sequencing. GBS analysis pipeline implementation, SNP filtering, and manipulation were described in Ferrão *et al*. (2017, 2018). SNPs with missing data over 50% were removed. Nonbiallelic SNPs and markers with minor allele frequency of less than 1% were also removed. After following the quality-control steps, a total of 56k markers were used for genomic prediction.

### Phenotypic data analysis

Phenotypic analyses were carried out with the following 2 main objectives: (1) determine the impact of number of harvests on the predictive ability and (2) estimate the predictive ability of trait stability metrics. To this end, we reasoned the following statistical approach to modeling the phenotypic data and use in subsequent genomic prediction models:

#### Number of harvests

To investigate the impact of number of harvests on genomic prediction, we considered 8 years of phenotypic data collected at the FEM location. The impact of including more harvest data in predictive studies was tested under 2 different scenarios of validation as described in Table 1. In both scenarios, the data was first divided into training and validation sets, in which the validation set was composed of phenotypes collected in 2014 and 2015, while the rest of the years were used for training. We used 2014 and 2015 as validation since both datasets were collected in mature coffee populations, with well-established genotypes in field conditions, and phenotypes collected after pruning.

**Table 1.** Statistical models. Models for estimating best linear unbiased estimates for different scenarios of year combinations.

Scenario	Year combination								Model^*a*^
I	**07**								Yikr_07_ = µ + G_i_ + B_k_ + e_ik_
II	**07**	**08**							Yijkr_07-08_ = µ + G_i_ + E_j07-08_ + B_k_|E_j_ + GE_ij_ + e_ijk_
III	**07**	**08**	**09**						Yijkr_07-09_ = µ + G_i_ + E_j07-09_ + B_k_|E_j_ + GE_ij_ + e_ijk_
IV	**07**	**08**	**09**	**10**					Yijkr_07-10_ = µ + G_i_ + E_j07-10_ + B_k_|E_j_ + GE_ij_ + e_ijk_
V	**07**	**08**	**09**	**10**	**11**				Yijkr_07-11_ = µ + G_i_ + E_j07-11_ + B_k_|E_j_ + GE_ij_ + e_ijk_
VI	**07**	**08**	**09**	**10**	**11**	**12**			Yijkr_07-12_ = µ + G_i_ + E_j07-12_ + B_k_|E_j_ + GE_ij_ + e_ijk_
VII							**14**	**15**	Yijkr_14-15_ = µ + G_i_ + E_j14-15_ + B_k_|E_j_ + GE_ij_ + e_ijk_

Where **Y_ijkr_** is the phenotype of the ith individual (I = 1,2…, *n*), of the jth environment (j = 1,2, …8), and of the kth block (k = 1,2,3); **µ** is the overall mean; **G_i_** is the genetic effect of individual; **E_j_** is the effect of environment; **B_k_|E_j_** is the block effect nested within environment; **GE_ij_** is the genotype by environment interaction and; **e_ijkr_** is the nongenetic residual error term.

After dividing our population into training and validation, we used our historical data to systematically include more years in the testing set and predict the validation set. For example, case I represents data collected only in 2007 and was used to predict phenotypes in 2014 and 2015. In the other extreme (case VI), we predicted 2014 and 2015 using the data collected from 2007 to 2012. All linear models were fitted using the ASReml R package (Butler *et al*. 2018), and the empirical best linear unbiased estimator (eBLUEs) for the genotype effects used in the subsequent genomic prediction analyses.

#### Trait stability

Trait stability was first measured using a multiplicative AMMI model, as follows:


$$Yij=\mu +gi+ej+\Sigma_{\left(n=1\right)}^{N}\lambda_{n}\gamma_{in}\delta_{\;jn}+\rho_{ij}$$

where: $Y_{ij}$ is the precorrected value of the *i*th genotype and *j*th environment, μ is the overall mean; **g_i_** and **e_j_** are the fixed effect of *i*th genotype effects; and *j*th environmental deviations, respectively, $\Sigma_{\left(n=1\right)}^{N}\lambda_{n}\gamma_{in}\delta_{\;jn}+\rho_{ij}$ is the GEI and *n* is the eigenvalue of *n*th principal component (PC), $\lambda_{n}$ is a singular value of the *n* axis in the PC axes (PCAs), $\gamma_{in}$, and $\delta_{\;jn}$ are the *i*th genotype and *j*th environment eigenvectors for the *n*th PC, respectively. $\rho_{ij}$ is the GEI residuals (Sabaghnia *et al*. 2008). GEI sum of squares was divided into interaction PCAs, which shows the portion of correspondence to a particular AMMI model.

The sum of squares of the PC (SPC) scores was used to calculate the AMMI stability index using the following equation: SPC = $\sum_{n=1}^{N}.\lambda_{n}^{0.5}\gamma_{in}$, where *N* is the number of PCs in the model for the *i*th genotype (Sabaghnia *et al*. 2008). The best model that described GEI was tested and selected using the F test (Cornelius *et al*. 1992). AMMI stability value (ASV) was estimated following: ASV $=\;\;\sqrt{\frac{SIPC1}{SIPC2}\;{\left(IPCA1\right)}^{2}+\;{\left(IPCA2\right)}^{2}}$, where SPC1 and SPC2 is the sum of squares for PCA1 and PCA2, respectively. A large absolute value of PCA indicates greater adaptability of a genotype to a specific environment. The lower the ASV, the greater the stability of a genotype in different environments (Oliveira *et al*. 2014). AMMI analyses were carried out in Agricolae R package v1.3–5 ([Bibr jkad062-B56][Bibr jkad062-B11]; de Mendiburu and de Mendiburu 2019).

A second popular approach to address GEI is based on linear regression models. A FW regression express the interaction in the form of heterogeneity of the slopes computed across individual genotypic performance under an environmental index. The inference is performed in two main steps: (1) estimate environmental effects from the environmental means using the main effects model and (2) estimate of intercepts and slopes of each genotype.


$$Y_{ij}=\mu +g_{i}+h_{j}+e_{ij}$$

$$Y_{ij}=\mu +g_{i}+h_{j}+b_{i}h_{j}+e_{ij}$$

where *μ* is the overall mean (intercept); **g_i_** is the main effect of *i*th genotype; **h_j_** is the main effect of the *j*th environment; **b_i_** + 1 is the change of expected individual performance per unit of change of **h_j_** (slope); **ĥ_j_** is the estimate of *j*th environment effect; **e_jj_** is the error term. The stability index of individual *i*th is the deviation of the corresponding individual slope (**b_i_**) from zero. Herein, we considered an individuals with “high stability” when its corresponding slope is close to zero—since they can keep their performance over different environment conditions. FW regressions were carried out using the FW R package (Lian and de los Campos 2016).

### Genomic selection

Whole-genome statistical models were used to predict coffee traits under different validation scenarios. First, we compared predictive models with different statistical assumptions. The Bayesian ridge regression (BRR) assumes that the marker effects are normally distributed with fixed variance, which could align better for trait predictions that follow this infinitesimal model. The BayesB model induces variable selection and assumes that most loci do not affect the phenotypic variation, a fact that aligns better for traits controlled by relatively few loci. Both models were fitted following linear regression, as proposed by Meuwissen *et al.* (2001): y=μ+Xβ+e, where y is an *n*-vector of phenotypes measured on *n*-individuals, after adjusting for fixed linear effects of blocks, years and locations, X is a matrix of genotypes measured; β is a *P*-vector of SNP effects to be estimated; and e is independent and normally distributed residuals. More details on the effect size distributions are discussed in Ferrão *et al*. (2018).

A second point of analysis in this study was how multi-population (MP) data could be integrated to better predict trait stability. To answer such a question, three statistical modeling approaches were implemented: (1) a single population (SP) model obtained by regressing phenotypes on markers separately in each coffee population; (2) a combined analysis where marker effects were treated as homogeneous across populations [across-population (AP) model]; and (3) a combined analysis where marker effects were assumed heterogeneous across populations, and marker x population interaction was accounted for. All the reference models were originally implemented by Lopez-Cruz *et al*. (2015) and are described in sequence.

#### SP model

A SP GS model is obtained by regressing the phenotype vector containing the trait records for each population, y_j_ = {y_ij_}, where i represent lines (individuals) and j represent populations, on markers using the following linear model:


$$y_{ij}=\mu_{j}+\Sigma_{k=1}^{p}x_{ijk}\beta_{\;jk}\;+\;e_{ij}$$

where y_ij_ is the phenotype of ith individual (i = 1, 2, …, *n*) of the jth environment (j = 1, 2) and kth markers (k = 1, 2, …, *P*); **µ_j_** is an intercept of j^th^ population, X**_j_** = {x_ijk_} is a marker matrix; **β_j_** = {**β_jk_**} is a vector of marker effects and **e_j_** is a vector of model residuals. X_1_ represents the number of reference alleles at a specific locus in the coffee genome and is coded as 0, 1, 2. Marker effects are the same, X_1_ = X_2_ = … X_s_, for lines evaluated in all environments in a full-factorial design. Marker effects and model residuals are assumed to be independent and normally distributed: **β_j_** ∼ *N*(0, I σ^2^**_β_**_j_) and e**_j_** ∼ *N*(0, I σ^2^**_e_**_j_).

#### AP model

In this model, marker effects are assumed to be the same across populations, such that: **β_1_** = **β_2_ = β** following the regression model described in matrix notation:


$$\left[\begin{array}{c} y_{1}\\ y_{2} \end{array}\right]=\left[\begin{array}{c} 1\mu_{1}\\ 1\mu_{2} \end{array}\right]+\left[\begin{array}{c} X_{1}\\ X_{2} \end{array}\right]\cdot \beta_{0}+e$$

where y is a vector of the adjusted phenotype of the individuals in populations I and II, X is the design matrix of marker effects for each population and $b_{0}$ is the vector of marker effects for population I and II. It was assumed that the error followed a normal distribution with mean zero and homogeneous variance.

#### MP model

In this model, marker effects are estimated in two components: a main effect across both populations and a specific effect computer per population. The final marker effect was computed after summed effects that are cluster-specific ($b_{1j}$ and $b_{2j}$ for population I and II, respectively) and markers that are common across clusters ($b_{0j}$, where j = 1, …, *P* indexes markers), as follow: $\;\beta_{1j}=b_{0j}+b_{1j}$ and $\;\beta_{2j}=b_{0j}+b_{2j}$ in populations I and II, respectively. The regression model for MP is presented below in matrix notation:


$$\left[\begin{array}{c} y_{1}\\ y_{2} \end{array}\right]=\;\left[\begin{array}{c} 1\mu_{1}\\ 1\mu_{2} \end{array}\right]+\left[\begin{array}{c} X_{1}\\ X_{2} \end{array}\right]b_{0}+\left[\begin{array}{c} X_{1}\\ 0 \end{array}\right]b_{1}+\left[\begin{array}{c} 0\\ X_{2} \end{array}\right]\cdot b_{2}+\ldots e$$

where y_1_ and y_2_ denote the adjusted phenotype of the individuals in populations I and II, respectively; $\mu_{1}$ and $\mu_{2}$ are population-specific intercepts; $b_{0}$ is the vector of marker main effects, $b_{1}$ and$b_{2}$ are vectors of marker population-specific interactions; and $e_{1}$ and$e_{2}$ are RES errors associated with both populations. It was assumed that the error followed a normal distribution with mean zero and homogenous variance.

### Cross-validation

Prediction accuracies of genomic selection models were accessed as the Pearson correlation between observed performance (BLUEs) and predicted GEBVs using: (1) marker effect estimated in one set of years to predict the genotype performance for other years (as indicated in Table 1) and (2) using cross-validations. Cross-validations were used to predict stability indices computed from AMMI and FW models in the stratified analyses and when populations were combined. In both cases, the phenotypic data was divided into training and validation sets using a 10-fold cross-validation approach, with 5 repetitions. All genomic predictions were implemented in the BGLR R package developed by Perez and de los Campos (2014). In the BGLR software, all hyperparameters for the models were implemented using the default settings (Perez and de los Campos 2014). The Markov Chain Monte Carlo for all models was set to 30,000 iterations and 5,000 burn-ins. The trace plot was checked to ensure convergence was reached.

The authors affirm that all data necessary for confirming the conclusions of the article are present within the article, figures, and tables.

## Results

### Descriptive summary

**Fig. 1.**
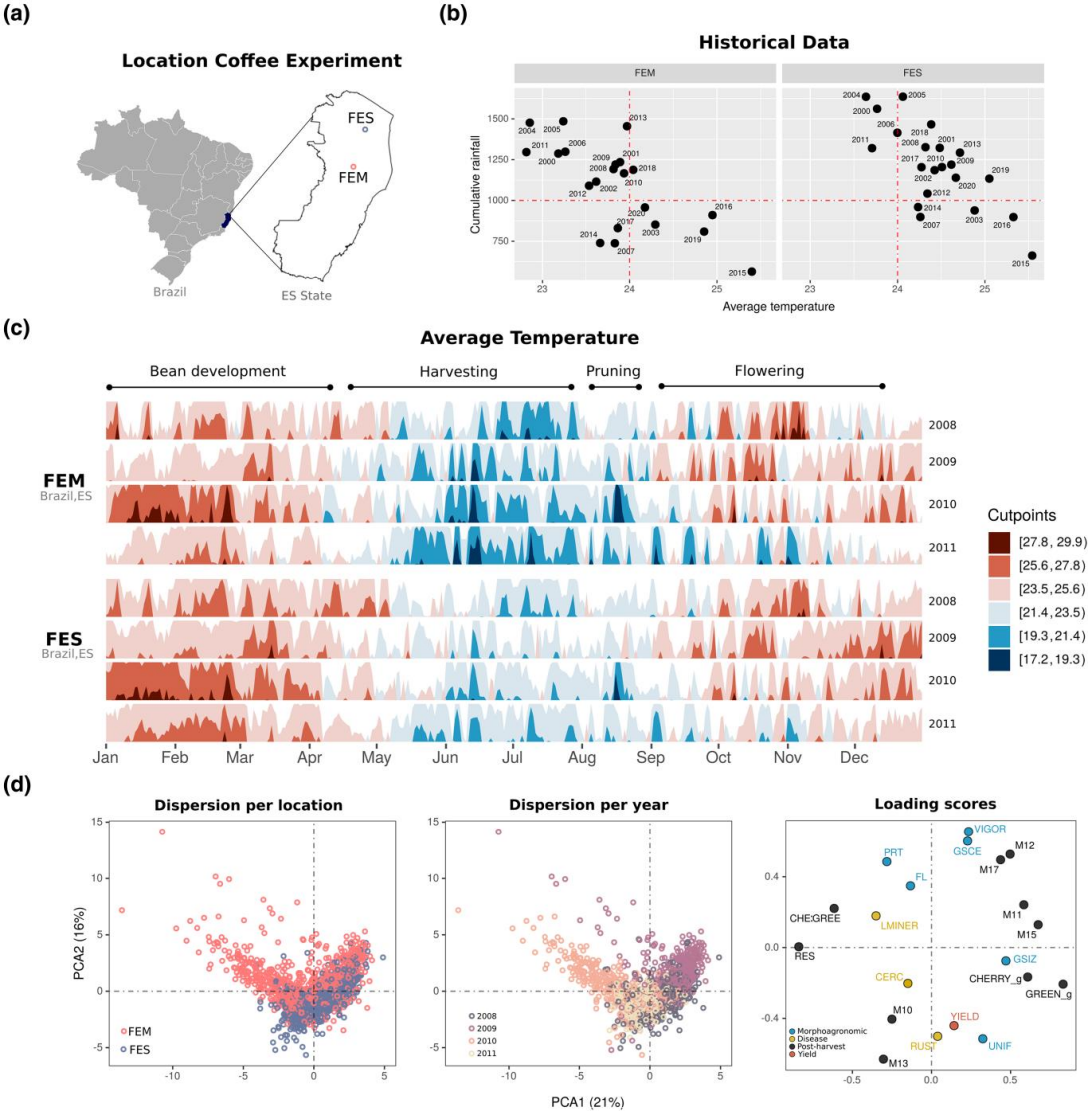
Descriptive analysis of the coffee population, traits, and environments. (a) Map of Brazil, the state (Espirito Santo State), and the locations (FEM and FES) where the coffee phenotypic data were collected. b) Historical data collected in the past 22 years for cumulative rain (mm) and average temperature (celsius) in both locations—reference lines indicating an rainfall of 1000 and temperature of 240 °C to help on the visual comparison of both sites. c) Average temperature collected in both locations from 2008 to 2011, years when the phenotypic data was collected for this experiment. d) PCAs based on 20 morpho-agronomic, disease resistance, postharvest, and yield traits. Genotypic dispersion is presented in the first 2 left plots, grouped by location and years, respectively. Loading scores are presented in the right plot with the relative importance of each trait.

This investigation was built on phenotypic evaluations performed in two Brazilian regions, representative of *C. canephora* production. Located in the Espirito Santo State (Fig. 1a), this region accounts for more than 30% of internal production. Although both sites are part of the same macro-region, growers have experienced different coffee performances, due to variable weather conditions. The historical weather data collected in both locations indicated more rainfall and higher temperatures in FES (Fig. 1b). For the 4-year harvests considered in this study, differences in temperature and rainfall between locations were significant (Fig. 1c and Supplementary Fig. 2). Using environmental covariables, we confirmed temporal and spatial variations by clustering the environments, based on average daily temperature, gradients summary, computed growing degree days, number of sunlight hours, and evapotranspiration from remote evaluations (Supplementary Fig. 3). We also clustered the genotypes based on the phenotypic metrics collected in both locations over the 4 years. From PCA, genotypes were primarily clustered in accordance with their locations, harvest years and population (Fig. 1d and Supplementary Fig. 4).

A second piece of relevant information extracted from PCA analyses is the relationship between coffee traits (Fig. 1d). Traits clustering in the same plane suggests high correlation—confirmed using Pearson's correlation analyses (Supplementary Fig. 5a and b). Among the traits collected in the *Premature* population, YIELD was significantly (*P* < 0.05) and positively correlated with VIGOR (0.55) and GSCE (0.64); and negatively correlated with RUST (−0.36), CERC (−0.24), and CHE:GREE ratio (−0.25). RUST, an important disease in coffee, was positively correlated with CERC (0.30), M15 (0.29) and M17 (0.21); and negatively correlated with UNIF (−0.26), VIGOR (−0.26), the postharvest traits M10 (−0.21), and RES (−0.28). In the *Intermediate* population, the correlation among traits was similar to what was obtained in the *Premature* population, but differences include significant negative correlations between YIELD with RES (−0.29), LMINER (−0.25), and GREEN (−0.20).

### Phenotypic performance and trait stability

The descriptive summary of phenotypic performances, heritability, and average stability using AMMI and FW models are presented in Table 2. In general, the *Intermediate* population showed superior phenotypic performance. Heritability estimates ranged widely among most traits in both populations, from 0.15 for CHE:GREE in the *Premature* population, to 0.92 for GSIZ in the *Intermediate* population. YIELD, an important coffee trait, showed large heritability values in both populations. Heritability values were also consistent across both locations.

**Table 2.** Descriptive analyses.

	Premature							Intermediate						
Trait	H2	lsMEANS		AMMI		FW		H2	lsMEANS		AMMI		FW	
		Mean	SD	Mean	SD	Mean	SD		Mean	SD	Mean	SD	Mean	SD
GSCE	0.75	6.47	1.02	0.33	0.21	0.00	0.50	0.76	6.60	0.99	0.47	0.26	0.00	0.40
FL	0.88	2.51	0.84	0.42	0.34	0.00	0.37	0.86	3.24	0.74	0.36	0.25	0.00	0.33
VIGOR	0.77	6.77	1.00	0.33	0.22	0.00	0.39	0.71	6.90	0.98	0.63	0.39	0.00	0.38
GSIZ	0.89	2.46	0.63	0.28	0.17	0.00	0.77	0.92	2.73	0.75	0.39	0.23	0.00	0.35
UNIF	0.53	1.81	0.67	0.23	0.16	−0.02	0.26	0.53	1.74	58.44	0.37	0.17	0.00	0.20
PRT	0.90	2.23	0.67	0.62	0.41	−0.04	0.65	0.92	2.05	0.70	0.52	0.29	0.00	0.52
CERC	0.57	2.90	1.34	0.67	0.63	−0.01	0.29	0.61	3.19	1.64	0.36	0.19	0.00	0.15
LMINER	0.32	3.34	0.84	0.32	0.17	−0.01	0.33	0.58	3.30	0.83	0.32	0.16	0.00	0.25
RUST	0.79	3.52	1.62	1.02	0.54	−0.01	0.74	0.74	4.22	1.77	0.58	0.32	0.00	0.40
CHERRY_g	0.81	434.45	110.21	4.66	3.11	0.01	0.42	0.73	458.73	458.73	3.73	2.48	0.00	0.39
CHE:GREE	0.15	2.45	0.69	3.30	6.35	0.00	1.96	0.23	2.44	0.51	2.48	3.96	0.00	1.06
GREEN_g	0.51	835.18	103.58	5.81	5.57	−0.01	1.23	0.76	840.91	112.09	8.41	9.78	−0.02	1.61
M10	0.81	34.90	25.75	4.44	3.53	0.02	1.18	0.75	32.89	21.11	2.34	1.13	0.00	0.51
M11	0.76	31.58	27.34	3.43	2.05	0.01	0.70	0.78	32.15	24.57	1.83	1.15	0.00	0.45
M12	0.78	8.13	12.47	1.95	1.56	−0.01	0.69	0.81	9.62	14.66	4.37	3.27	0.00	0.62
M13	0.82	70.36	43.71	4.72	2.86	−0.01	0.52	0.87	72.01	47.16	3.26	1.86	0.00	0.35
M15	0.89	80.99	54.33	5.73	3.47	−0.04	0.81	0.67	87.88	51.11	6.61	4.14	0.00	0.53
M17	0.86	19.47	37.65	5.41	3.37	0.00	1.07	0.86	18.94	34.12	5.50	4.45	−0.01	0.88
RES	0.90	54.35	62.55	4.72	2.86	0.01	0.55	0.69	46.42	58.44	6.12	4.06	0.00	0.46
YIELD	0.76	20.69	12.12	1.25	0.65	−0.02	0.41	0.73	21.12	12.96	1.37	0.75	0.00	0.30

Summary of heritability (H2) values. Mean and SD of the adjusted phenotypic values (lsMEANS). Mean and SD of the additive main effect computed using multiplicative interaction stability (AMMI index). Mean and SD values associated to FW regression stability coefficients. Values were computed for all coffee traits evaluated in the premature and intermediate populations.

GEI was first noticed by crossover interactions observed when the relative difference between genotypic means were investigated over the environments (Supplementary Fig. 6). Trait stability was computed using both AMMI and FW approaches (Supplementary Fig. 3a and b). The number of PCs used in estimating AMMI stability was uniform for all traits, with the first 2 PCs retaining a moderate proportion of variances (∼ 50%). The average AMMI stability for all traits based on 2 PCs ranged from 0.23 for UNIF to 5.81 for GREEN_g in the *Premature* population, while it ranged from 0.32 for LMINER to 8.41 for GREEN_g in the *Intermediate* population. When evaluated in terms of regression analyses, the mean for the FW stability index was approximately zero for all traits in both populations, and the standard deviation showed heterogeneity among slopes. We further quantified the importance of GEI effects by estimating variance components for all traits, and the percentage of GEI variation explained by the first two components using AMMI models (Supplementary Table 1). Estimated genotype, environment, and GEI effects for all traits were significant (*P* < 0.001) across populations, confirming the relevance of temporal and spatial variation in *C. canephora*.

The linear relationship between phenotypic performance and environmental stability projects future improvements in selecting individuals with high performance and desirable stability. Non-significant associations were observed, which were more related to the AMMI estimates. In particular, a low correlation (0.09) value was observed between YIELD and the AMMI metric computed for the *Intermediate* population. In contrast, RUST had a negative and low correlation (−0.11) for the AMMI metric only in the *Premature* population. Based on the AMMI results, consistent low correlations observed across both populations were observed only for VIGOR, CHERRY_g, M13, and RES traits. When contrasted with the computed FW slopes, Person's correlation between phenotypic performance and stability metric for most traits were significantly associated. The only trait that was consistently scored as statistically nonsignificant across both coffee populations was the GREEN_g trait.

#### Genomic prediction

For genomic prediction, we tested three main scenarios addressing (1) the relevance of testing genomic selection models with different statistical assumptions; (2) the importance of collecting multiple-year harvest data; and finally (3) the predictability of trait stability.

**Fig. 2.**
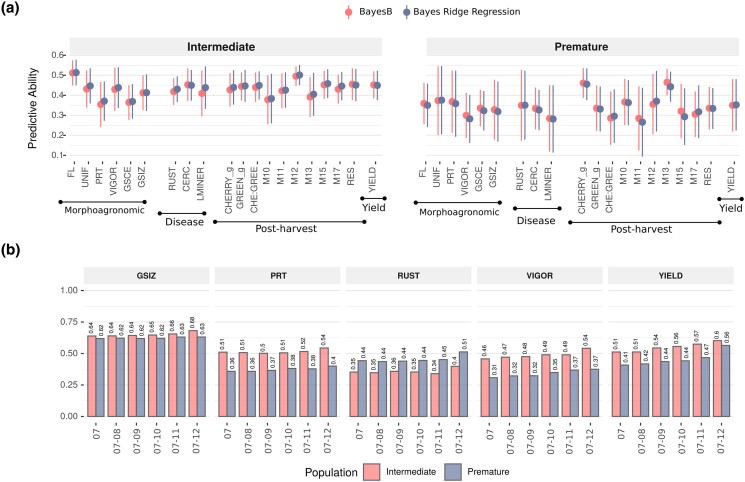
Genomic prediction of coffee traits in different validation scenarios. a) Predictive ability computed using Bayes Ridge Regression (BRR) and BayesB for 20 coffee traits encompassing 4 main categories: morphoagronomic, disease resistance, postharvest, and yield traits. b) Predictive ability measured using the BRR model on historical data collected in the FEM location. Predictive models were trained using a varying set of years, incremented systematically, in the training set to predict phenotypes evaluated in 2014 and 2015.

When contrasting the predictive abilities of BRR and BayesB models, both models showed similar performance across all traits in both populations (Fig. 2a). The closest findings were in average predictive abilities for different traits and locations, which ranged from 0.26 for the M11 trait evaluated in the *Premature* population to 0.51 for the FL trait in the same population, in both models. The most extreme differences between models were observed for the M13 trait measured in the *Premature* population and the LMINER trait collected in the *Intermediate* population. Average prediction accuracies were higher generally in the *Intermediate* population (0.433 vs 0.341). We also observed great variation across the traits. For example, in both populations, large predictive abilities were noted for the postharvest traits; in particular, CHERRY_g, M12, and M13 traits.

The second question underlies the effectiveness of increasing the number of years as a means of improving the predictive ability of a model. Overall, in both populations, prediction accuracies for all traits increased when more years were included in the training set. Results are better illustrated in Fig. 2b, where some key coffee traits were selected to show some trends. For the GSIZ, PRT, and RUST traits, no clear evidence of prediction accuracy improvement was observed after two years of phenotyping. Comparatively, VIGOR and YIELD were traits that benefited from the addition of more historical data. In addition, the accuracy of best genotype selection increased for yield with increasing number of years (Supplementary Fig. 7).

**Fig. 3.**
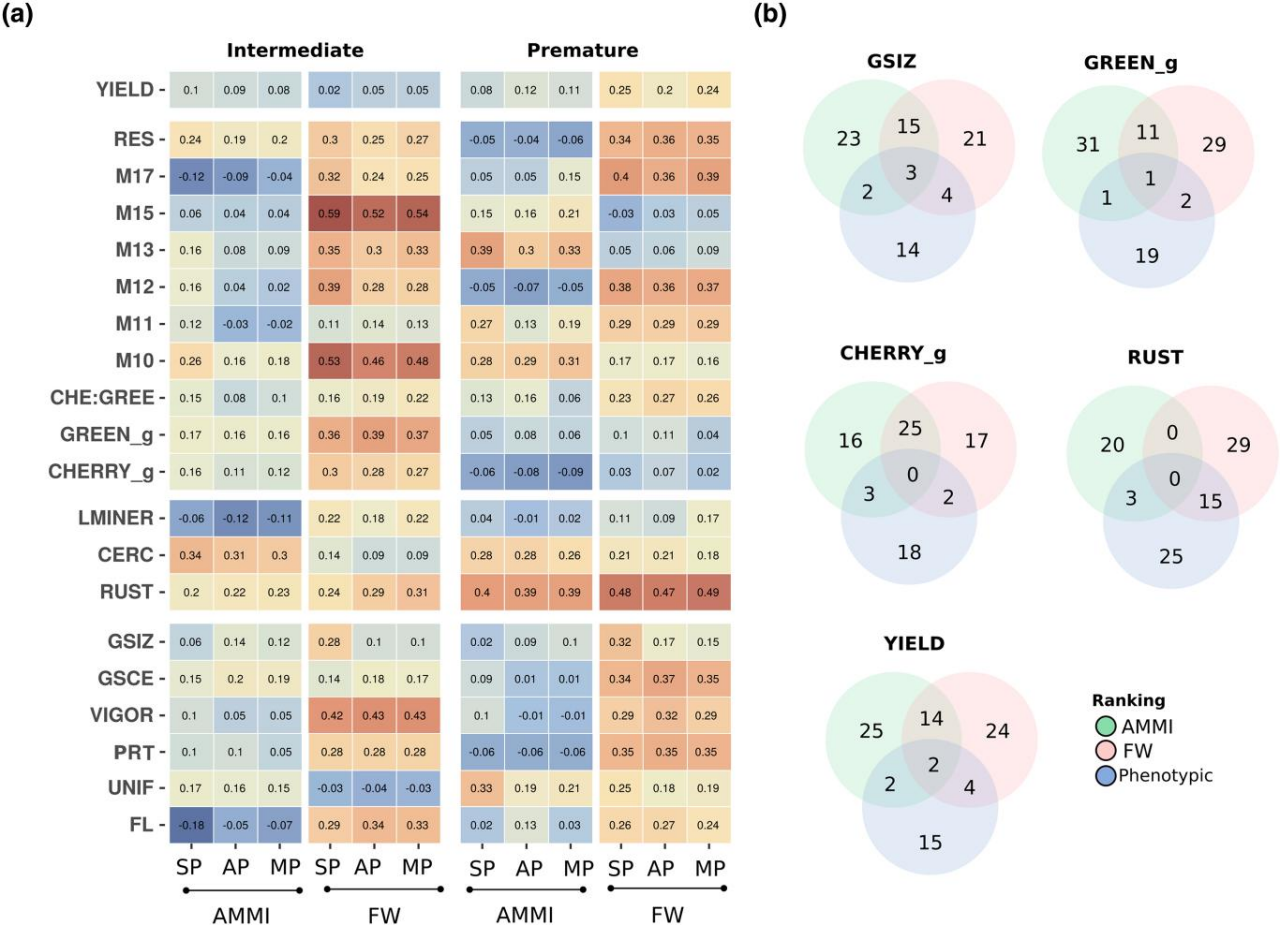
Genomic prediction of trait stability in coffee. a) Predictive ability measured for stability metrics computed using the AMMI and FW regression using 3 competitive whole-genome statistical models: SP, AP, and MP. b) Venn diagrams between trait performance and stability for best genotypes. Genotypes with best 10% for trait performance, top 20% for AMMI stability (lowest ASV), and top 20% for FW stability (absolute b ∼ 0).

In the last set of scenarios, the prediction accuracy for stability was evaluated. In view of statistical modeling, we tested the importance of performing predictions by gathering phenotypic data from multiple breeding populations and testing it in the form of three complementary statistical models: SP, AP, and MP. Overall, the average prediction accuracy for *Premature* population trait stability was slightly better than observed in the *Intermediate* population. When FW and AMMI metrics were contrasted, trait stability measured by FW regressions was more predictable using genomic information than AMMI metrics. In view of model parametrization, we could not observe larger differences between the three different models, suggesting that a more parsimonious implementation (SP model) is sufficient to maintain good predictive abilities across all traits in both populations (Fig. 3a).

Our last research question addressed whether genotypes with high phenotypic performance and stability across years and locations could be simultaneously selected. Both the summary of average stabilities (Table 2) and Pearson's correlation (Table 3) indicated significant correlations between stability metrics and phenotypic performance. Generally, GSCE, M12, and M17 showed significant (*P* < 0.01) positive correlations (ranging from 0.44 to 0.96) between trait values and stability estimated with AMMI and FW in both populations. Herein, the top 10% of best genotypes for each trait were ranked and compared to the top 20% of genotypes selected from the AMMI stability (lowest ASV) and the top 20% for FW stability (absolute b ∼ 0). In Fig. 3b, the overlap between the top genotypes in the form of Venn diagrams for some key traits is presented. From the results, the intersection of both stability models was able to identify top-performing genotypes for GSIZ (3), GREEN_g (1), and YIELD (2). Either the AMMI or the FW model was able to select top-performing genotypes for GSIZ (26%), GREEN_g (13%), CHERRY_g (22%), RUST (13%), and YIELD (26%). Generally, the FW approach was able to identify more stable genotypes with high phenotypic performance, when compared to the AMMI results.

**Table 3.** Correlation results.

Trait	Premature		Intermediate	
	AMMI	*FW*	AMMI	*FW*
GSCE	−0.21^*a*^	−0.37^*c*^	−0.18^*a*^	0.19^*a*^
FL	0.01ns	−0.25^*b*^	0.19^*a*^	0.01ns
VIGOR	−0.17ns	−0.24^*a*^	−0.15ns	0.39^*c*^
UNIF	0.33^*c*^	0.36^*c*^	−0.11ns	0.67^*c*^
GSIZ	−0.32^*c*^	0.23^*a*^	−0.08ns	0.26^*a*^
PRT	−0.07ns	−0.17^*a*^	0.15ns	−0.30^*b*^
CERC	0.48^*c*^	0.29^*b*^	0.07ns	0.24^*b*^
LMINER	0.02ns	0.30^*b*^	0.24^*b*^	0.28^*b*^
RUST	−0.11ns	0.64^*c*^	−45.44^*c*^	0.66^*c*^
CHERRY_g	−0.05ns	−0.30^*b*^	0.13ns	0.36^*c*^
CHE:GREE	0.74^*c*^	0.79^*c*^	0.46^*c*^	0.54^*c*^
GREEN_g	−0.32^*c*^	−0.13ns	0.06ns	−0.14ns
M10	0.43^*c*^	0.01ns	0.31^*c*^	0.39^*c*^
M11	0.14ns	0.29^*a*^	0.25^*b*^	0.05ns
M12	0.56^*c*^	0.96^*c*^	0.44^*c*^	0.88^*c*^
M13	0.07ns	0.49^*c*^	0.04ns	0.66^*c*^
M15	0.28^*b*^	0.22^*a*^	−0.02ns	−0.15ns
M17	0.68^*c*^	0.87^*c*^	0.55^*c*^	0.81^*c*^
RES	0.18ns	0.51^*c*^	0.10ns	0.66^*c*^
YIELD	0.20^*a*^	0.43^*c*^	0.09ns	0.51^*c*^

Correlation between the phenotypic performance and stability estimates. Linear relationship measured as Pearson's correlation between the phenotypic performance and environmental stabilities computed using AMMI model, and FW regression.

Indicates a significant correlation at *P* < 0.05.

Indicates a significant correlation at *P* < 0.01.

Indicates a significant correlation at *P* < 0.001.

## Discussion

### On the relevance of molecular breeding in *Coffee canephora*

Coffee is a universal beverage that drives a global industry and supports the economy of several developing countries. It is estimated that more than 100 million farmers contribute to the coffee chain, benefiting from crop profitability and sustainability in many parts of the world. In this scenario, Brazil has an important role to play being the world's largest producer and exporter of coffee (Volsi *et al*. 2019; World Coffee Portal 2021). It is estimated that one-third of the international production is currently supported by the Brazilian coffee supply chain, in which *C. canephora* beans correspond to more than 45% of national production (ICO 2020; Venancio *et al*. 2020). Also called Robusta and Conilon coffee, *C. canephora* stands out for being denser, less sweet, presenting less acidity, and having an accentuated aroma. Beans are used primarily by the industry in instant coffee, espresso, and as a filler in certain blends of ground coffee. More productive and resilient to biotic and abiotic stresses than *C. arabica*, Robusta/Conilon coffee has potential in coffee breeding, especially due to the projected conditions under climate change (Gomes *et al*. 2020; Venancio *et al*. 2020).

Despite the importance, genetic improvements in *C. canephora* have experienced slow progress in the last decades. Mostly focused on recurrent selection, breeding cycles in coffee can take up to 6 years. This is due to a long juvenile stage during coffee development, difficulty in data collection, management of large experimental designs, biannual effects, and the lack of information about the genetic architecture of complex traits (Souza *et al*. 2019; Alves *et al* 2020). In this regard, modern breeding programs have incorporated genomic prediction in their framework, with the advantage that genetic gains can be maximized by shortening breeding cycles and increasing prediction accuracies (Grattapaglia *et al*. 2018; Souza *et al*. 2019). In coffee, genomic selection has been tested for different traits and scenarios only recently. Overall, all investigations have confirmed its potential when compared to phenotypic and/or pedigree analyses (Ferrão *et al*. 2017, 2018; Souza *et al*. 2019). However, the referenced studies did not address an important issue underlying coffee breeding: the use of genome-base models to select coffee materials that combine high phenotypic performance and trait stability.

### Temporal and spatial variation are important GEI components in coffee

Our first contribution in this study relies on investigating a diverse set of coffee traits. Correlation analyses highlighted some important trends. For example, in both populations, a negative and significant correlation between yield and disease-resistance traits was observed. We also observed a positive correlation between yield and postharvest traits, suggesting that future improvements in quality could be achieved without compromising production. In terms of future genetic improvements, moderate-to-high heritability values were calculated and observed for most traits. Such facts indicate that genetic progress can be made for the traits under selection. Corroboratively, Omomdi (1994), Olika *et al*. (2011), and Getachew *et al*. (2017) also observed medium-to-high heritability for coffee bean characteristics, while the authors reported moderate heritability for coffee yield and disease severity.

When genotype-by-environment was explored, differences in the genotypic, environmental, and GEI variances were observed for all coffee traits evaluated in both populations. Environmental variance was an important contributor to total phenotypic variance, indicating the importance of temporal and spatial variation in coffee. While both FEM and FES locations are within the same -region, heterogenous components associated with GEI can be attributed to dynamics of weather conditions and disease incidence observed across both sites. Given the importance of GEI effects, it is a reasonable argument that different cultivars could be deployed per target region. Although valid, the dynamic of coffee production in Brazil—mostly supported by public breeding programs—indicates that the use of common cultivars in different regions can ultimately unify the management of the crop. Such reality offers opportunities for breeders to select superior and stable coffee genotype(s) across locations and years.

To quantify the importance of GEI, we first observed substantial crossover genotypic ranking when the relative difference between genotypic means over different environments was studies. In the sequence, we used AMMI models and observed that interaction variation accounted by PC1 and PC2 was above 50% for most of the traits in both populations. We complemented our investigation using FW regression models. At the genotypic level, we classified some genotypes with approximated absolute phenotypic stability for most traits. This implies that a good number of coffee genotypes can be selected for poor and favorable environments. For the specific goal raised in this study, coffee breeders are looking for genotypes that combine wide adaptability and high yield, in combination with other favorable traits. Interestingly, most traits demonstrated significant correlations (0.18–0.96) between phenotypic performance and trait stability estimated with either AMMI or FW in both populations. Hence, it is reasonable to suggest that phenotypic performance and trait stability can be simultaneously selected for the target environments evaluated in this study.

### A diverse set of coffee-related traits can be predicted using whole-genome statistical models

Our second contribution relies on using genomic selection in a practical breeding program. To our knowledge, there are no studies in coffee addressing predictive performances in a such diverse set of traits, evaluated in multiple coffee populations and environments. Therefore, when morpho-agronomic, disease resistance, yield, and postharvest traits were investigated, we observed low-to-moderate (0.28–0.51) predictive abilities. The average prediction accuracy was slightly higher for traits evaluated in the *Intermediate* population, which could be due to the differences in genetic architecture and expression of phenotypic characteristics in different populations. In general, the high accuracy of GEBV obtained from this study indicates that GS could be effective for all coffee traits.

Critical at the implementation level, when a new set of traits are investigated for genome-based prediction, an important question is what statistical method might better describe the relationship between the phenotypic and genetic variation (Ferrão *et al*. 2018). Several analytical approaches have been proposed in the specialized literature and here two contrasting models were compared: BRR, which assumes that marker effects are normally distributed with fixed variance, similar to the Fisher's infinitesimal model, and BayesB, which assumes that most loci have no effect on the phenotypic variation and therefore traits are genetically controlled by relatively few loci. Although conceptually different, we observed similar predictive performance across the traits, a fact that was also reported in other studies (De Almeida Filho *et al*. 2016; Ferrão *et al*. 2018; Rios *et al*. 2021).

Translating insights from the genomics into breeding decisions also brings complications on how and when to recalibrate the predictive models. The conventional use of 4 harvests in coffee research has biological and logistics arguments. From a biological standpoint, conventional phenotypic studies relying on previous repeatability coefficients that have reported a minimum number of 4 harvests to keep accurate results (Ferrão *et al*. 2019). From a logistics level, most public breeding programs have experienced seasonal labor and resource shortages in routine activities. When this is the case, using a minimum of four seasons for data collection is indeed an alternative to reduce biases if an assessment year is lost due to resource scarcity. However, with selection framed in terms of genomic prediction, can we optimize the number of harvests and hence reduce labor?

To answer this question, we designed a validation scenario where we systematically included more years to predict an independent data set. Herein, we hypothesize a situation where predictive models need to be recalibrated and breeders need to decide the number of harvest years for training their models. In general, our results suggested that reasonable prediction accuracies can be achieved when the data from two harvest years are collected. Arguably, traits like YIELD and VIGOR were more sensitive to the number of harvests. However, it is debatable if the costs and time necessary to perform long evaluation cycles are worth marginal gains in accuracy. On the other hand, breeders could indeed focus on reducing the breeding cycles, and saving space and time by selecting materials in earlier stages—a fact that will also maximize the genetic gains when expressed in terms of cycle length. Furthermore, it has been demonstrated that accelerating the breeding cycle can have a higher impact than increasing the accuracy in perennial species such as coffee (de Almeida Filho *et al*. 2016; Calleja-Rodriguez *et al*. 2020; Ferrao *et al*. 2021; Rios *et al*. 2021).

Remarkably, the use of a smaller number of harvests for training genomic prediction models does not invalidate the importance of using multiple harvests and MET in later stages of coffee breeding programs. Coffee breeding initiatives are to be encouraged to use such a strategy for variety deployment. Our results are shedding light on the possibility of reducing labor and costs related to training predictive models, by envisioning future rapid breeding cycles, where predictive models need to be consistently recalibrated.

### Accurate prediction for stability metrics opens new possibilities for selection in coffee

Our last contribution focuses on the use of genome-based methods to predict stability metrics. After estimated stability metrics using AMMI and FW methods, predictions were made using BRR models. For all traits, we observed low-to-moderate prediction accuracies. Similar results were also reported by Huang *et al*. (2016), which predicted stability metrics in a wheat breeding program. In common, both studies showed results with lower values associated with AMMI stability prediction when compared to FW regression. The GEI pattern captured using AMMI models and retained by the first two PCs, for some complex traits, might explain large portions of the interaction that could eventually reduce the predictive ability. Notably, among the traits evaluated here, we noticed that disease-related traits showed very consistent results and high stability prediction values in both populations for both methods. For other traits, important differences across populations were recorded. For example, stability associated with YIELD showed promising values only for the *Premature* population. Such a discrepancy can be associated with differences in allele frequency and linkage disequilibrium between SNPs and quantitative trait loci in both populations.

Aiming to improve the ability to predict trait stability, an assessment was conducted to determine if incorporating the information of multiple populations would affect predictive ability. The rationale behind this approach is that a larger training population size could leverage statistical power, and therefore genetic information could be borrowed across related populations. To test it, we used 3 statistical approaches to address marker-by-environment interactions. The SP approach refers to the regression of stability metrics on markers separately in each population. Then, when both populations are combined—without accounting for eventual interactions—we tested the AP method assuming that marker effect is homogeneous across populations. Finally, the MP model is a hybrid between the AP and SP models and hence accounted for common and specific marker effects across the populations. Similar approaches were reported in other crops, with relative success for predicting genotypes across environments and populations (Yao *et al*. 2017; Veturi *et al*. 2019).

The average prediction accuracies across traits and models showed slight superiority for SP models when predicting metrics associated with AMMI, while AP models yielded better predictive accuracies for the FW methods. Regarding our research questions related to the relevance of including multiple populations for coffee, we observed limited benefits when populations were combined (AP and MP), similar finding was also observed in cattle and corn (Hayes *et al.* 2009; de Los Campos *et al*. 2015). The similarity between predictions from SP and AP could be due to the lack of genetic correlation or relatedness between both coffee populations, a fact that could restrict the information borrowed between individuals across populations. Altogether, our findings are indicating that stability is a predictable metric and hence could be inserted into a genomic prediction framework. Particularly, more parsimonious models with populations trained separately is a valid alternative to combining predictive ability and computational cost.

### Selecting coffee genotypes with high phenotypic performance and environmental stability

Ultimately, the aim of this study was to translate insights from genomic prediction into genetic resources that can be used by coffee breeders. It was found that GEI associated to temporal and spatial variation contributes as much as genotypic effects to variance for most traits. Across environments, change in the rank of coffee genotypes for yield is common, indicating that individual genotypes were generally not high-yielding in all combinations of harvest-years. Other traits also showed similar patterns of rank-changing, a fact that highlights the complexity of GEI effects in coffee. Our results suggested that genomic prediction can be used for measure trait stability of some traits. Differences between AMMI and FW results indicate that GEI is assessed differently in both methods; similar findings was also noticed by Huang *et al*. (2016) in wheat. The task of selecting genotypes that combine phenotypic performance with environmental stability relies on gathering information from GEBVs and stability metrics associated with the traits. A correlation between both metrics was noted, which sheds light on the importance of targeting genotypes with high performance and less susceptibility to environmental variations. Recently, Olivoto *et al*. (2019) formalized a genotypic index whereby BLUPs and stability coefficients were considered. Namely, the Weighted Average of Absolute Scores algorithm models the phenotypic performance as a function of the stability computed in different environments. Herein, we opted to rank the genotypes using an agnostic approach, by looking for overlaps between top genotypes ranked via genomic prediction, FW, and AMMI metrics. In the context of practical implementations, breeders can rely on using selection indexes to weigh the importance of each trait and its stability for future decisions.

### Conclusion

Regarding our main research questions, we can conclude that (1) in general, good predictive abilities could be found when data was collected in 3 years, an information that opens new avenues to reducing coffee breeding cycles, reduce costs, and ultimately leverage genetic gains; (2) stability metrics could be predicted, in particular, using the FW regression; and finally (3) more parsimonious models, with populations trained separately, is a valid alternative to combine predictive ability and computational cost. Overall, the methods and approaches we used here allow for the simultaneous selection of genotypes with large GEBVs and stability across target environments. It should be emphasized that such findings are restricted to 2 populations evaluated in very specific locations and timeframes, a fact that could narrow down our conclusions. For a big picture on the genetic architecture associate to stability in coffee traits, we emphasize the need for experiments that contemplate a larger number of representative (and contrasting) site, evaluated over multiple years.

## Supplementary Material

jkad062_Supplementary_Data

## Data Availability

Genotypes in the study belong to the germplasm collection and breeding program of the Incaper institution (ES, Brazil). Phenotypic data used are reported in the supplementary information. Genomic information used in the genomic selection analyses are deposited in Dryad https://doi.org/10.5061/dryad.1139fm7. The R code for running the stability analysis is provided here: https://github.com/Pauliben/Prediction-of-Trait-Stability-in-Coffee- Supplemental material available at G3 online.
