# Supplementary material for: Genomic selection for genotype performance and environmental stability in Coffea canephora
Source: G3 (Bethesda). 2023 Mar 22;13(6):jkad062. doi: 10.1093/g3journal/jkad062 (PMC10234400; doi:10.1093/g3journal/jkad062)
Supplement: jkad062_Supplementary_Data [file jkad062_supplementary_data.zip › jkad062_Supplementary_Data.docx]

**Genomic selection for genotype performance and environmental stability in *Coffea canephora***

**Supplementary Results**

**
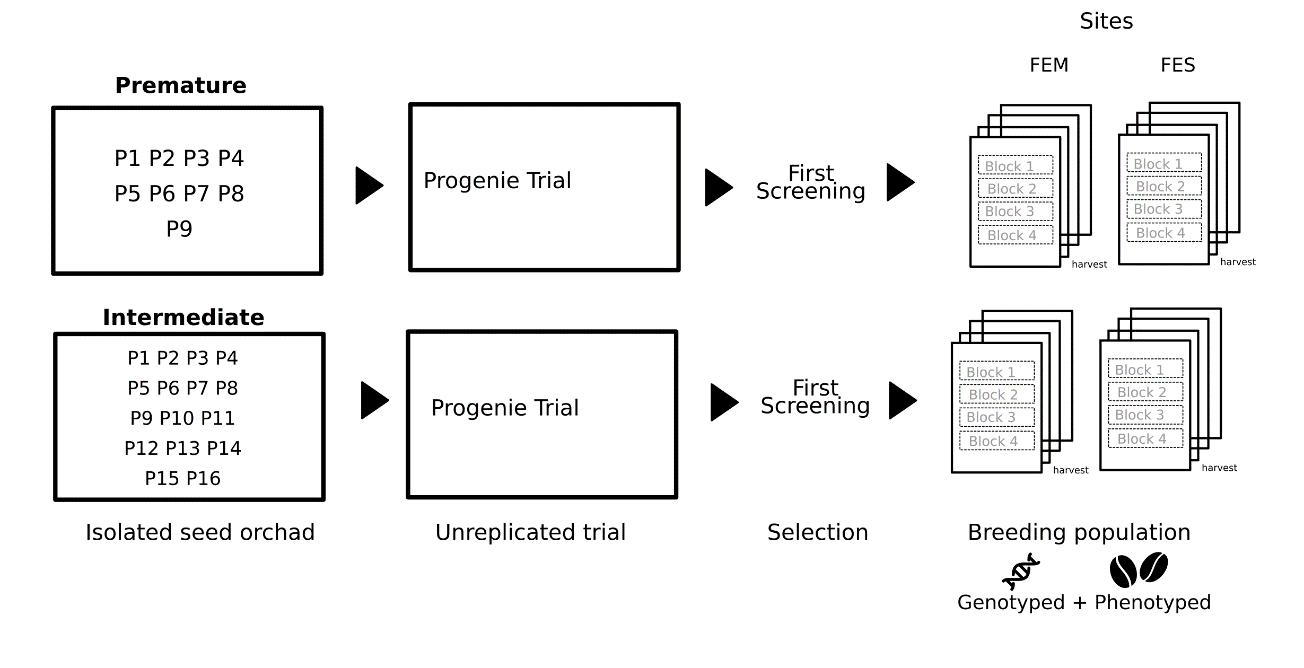
**

Figure S1. The populations used in this study were generated as part of the coffee breeding program at the Instituto Capixaba de Pesquisa, Assistência Técnica e Extensão Rural (Incaper), in partnership with Embrapa Café, Brazil. Since 1985, the institution has an active breeding program of C. canephora and among of thousands of genetic materials maintained in the germplasm collection, we focused on the selection of 9 and 16 superior founders to compose two complementary breeding populations for genetic analyses. These genotypes were visually selected due their outstanding agronomical traits, including high production, resistance to rust disease (Hemileia vastatrix), and, in particular, different maturity date. In 2001, these genotypes were included in two isolated seed orchard for open pollination creating two base population (Premature and Intermediate) for a recurrent selection breeding program. After one cycle of recombination, seeds were derived from each maternal plant, planted in a non-replicated field trial, and submitted to a first visual screening for four harvests. Best progenies were selected based on their yield production, resistance to rust and drought tolerance. In 2006, the best progenies were cloned and assigned to a randomized complete block design, with three replication, five plants per plot, and evaluated for multiple harvest-production years and two representative locations (FEM and FES).

**
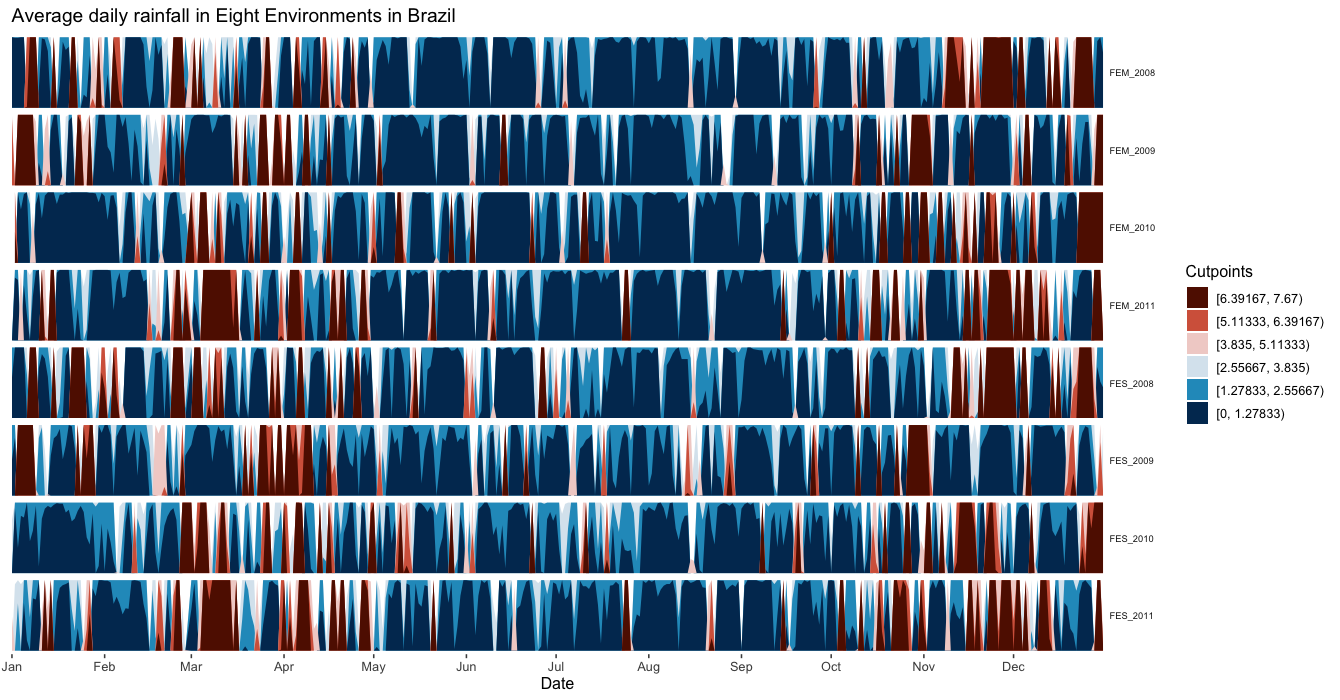
**

Figure S2. Average rainfall collected in both locations from 2008 to 2011, years when the phenotypic data was collected for this experiment


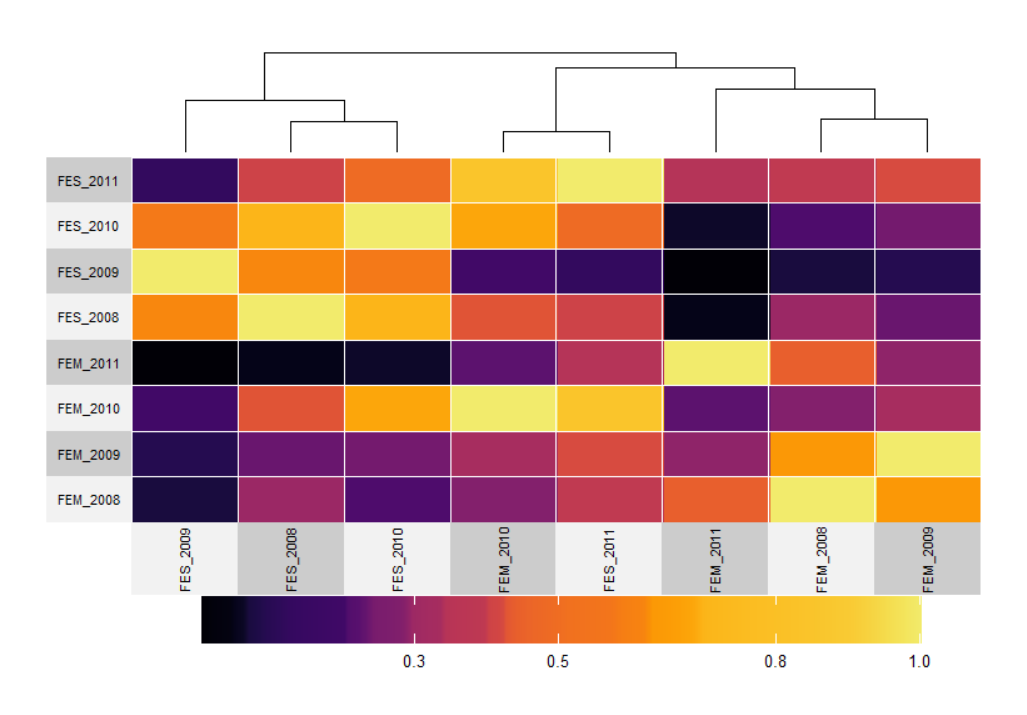


Figure S3. Visualized dissimilarity between eight environments in coffee METs in Brazil. Environmental similarity matrix using growing degree days, duration of sunshine hours and evapotranspiration. The matrix was computed using Gaussian kernel method premised on the envirotypes observed from remote information.


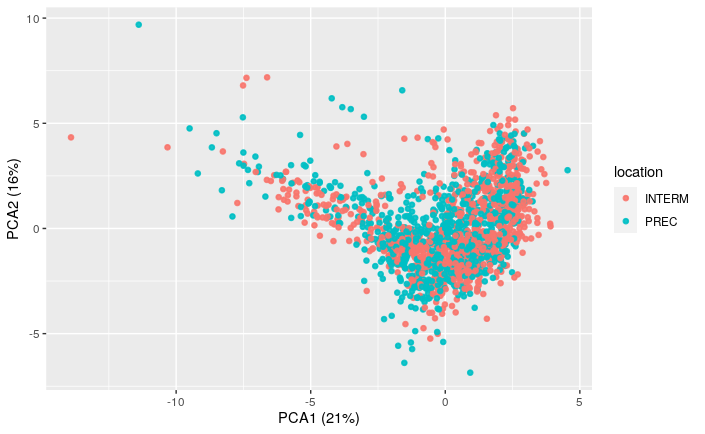


Figure S4. Principal component analyses PCAs based on 20 morpho-agronomic, disease resistance, post-harvest and yield traits. Genotypic dispersion was grouped by population

(a) (b)


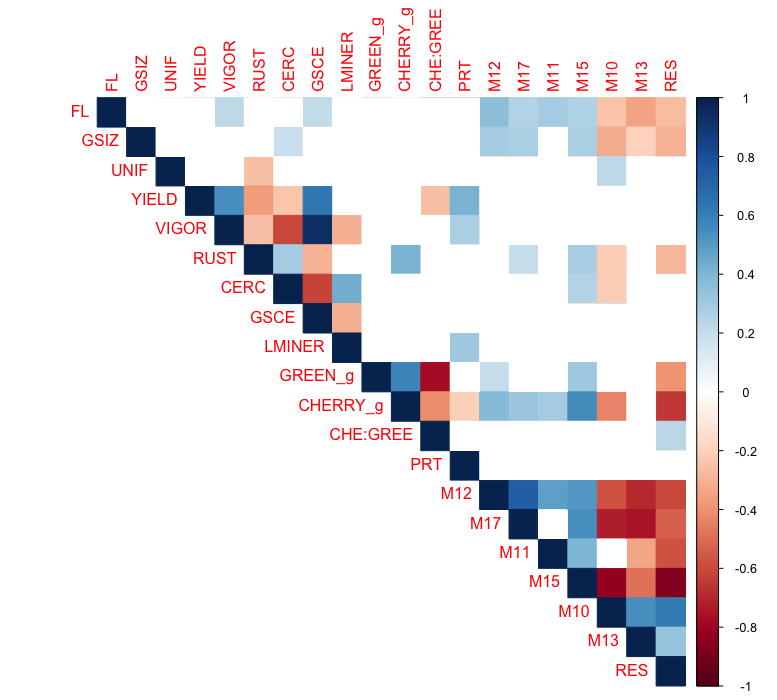

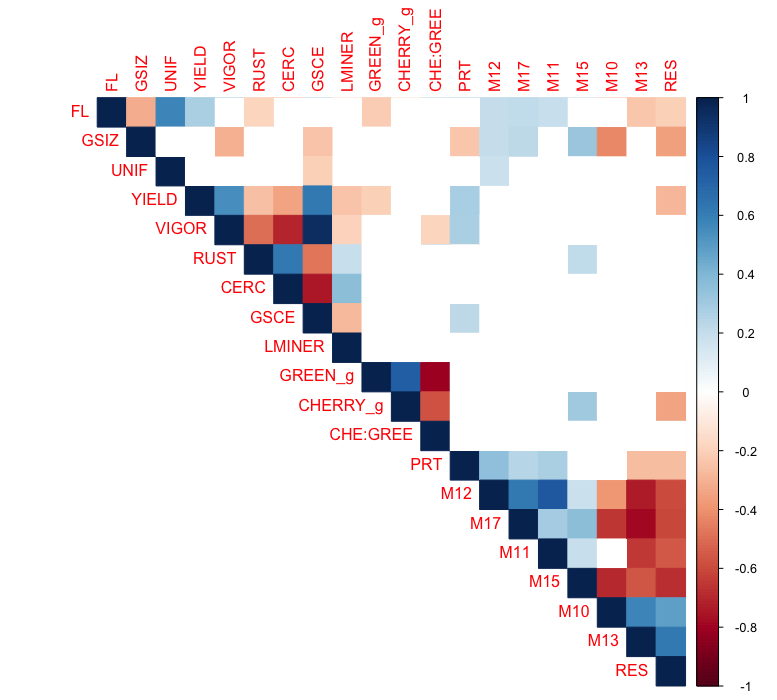


Figure S5. Pearson’s correlation among selected coffee traits based on least square means of each genotype in (a) premature (b.) intermediate populations.


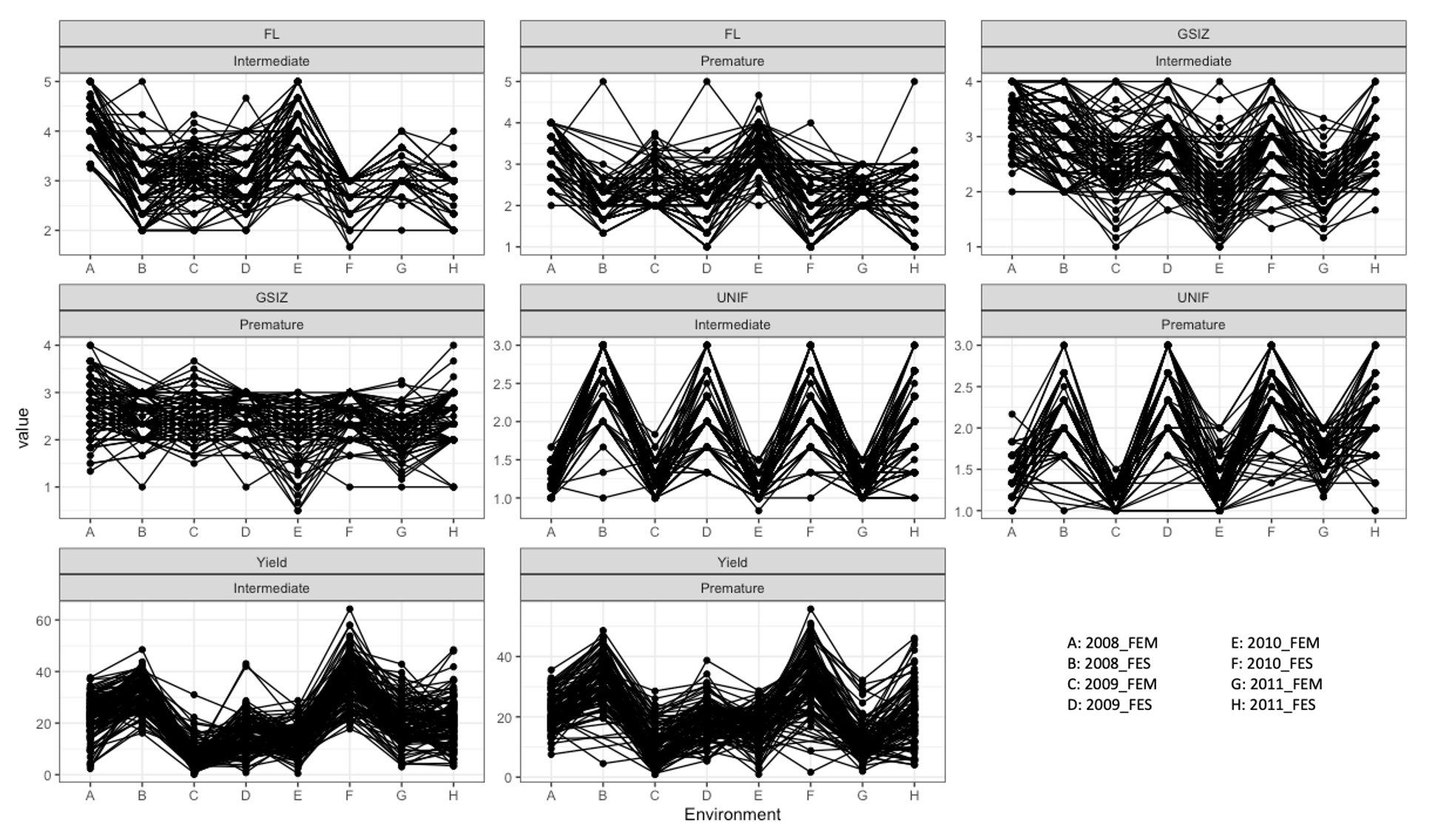


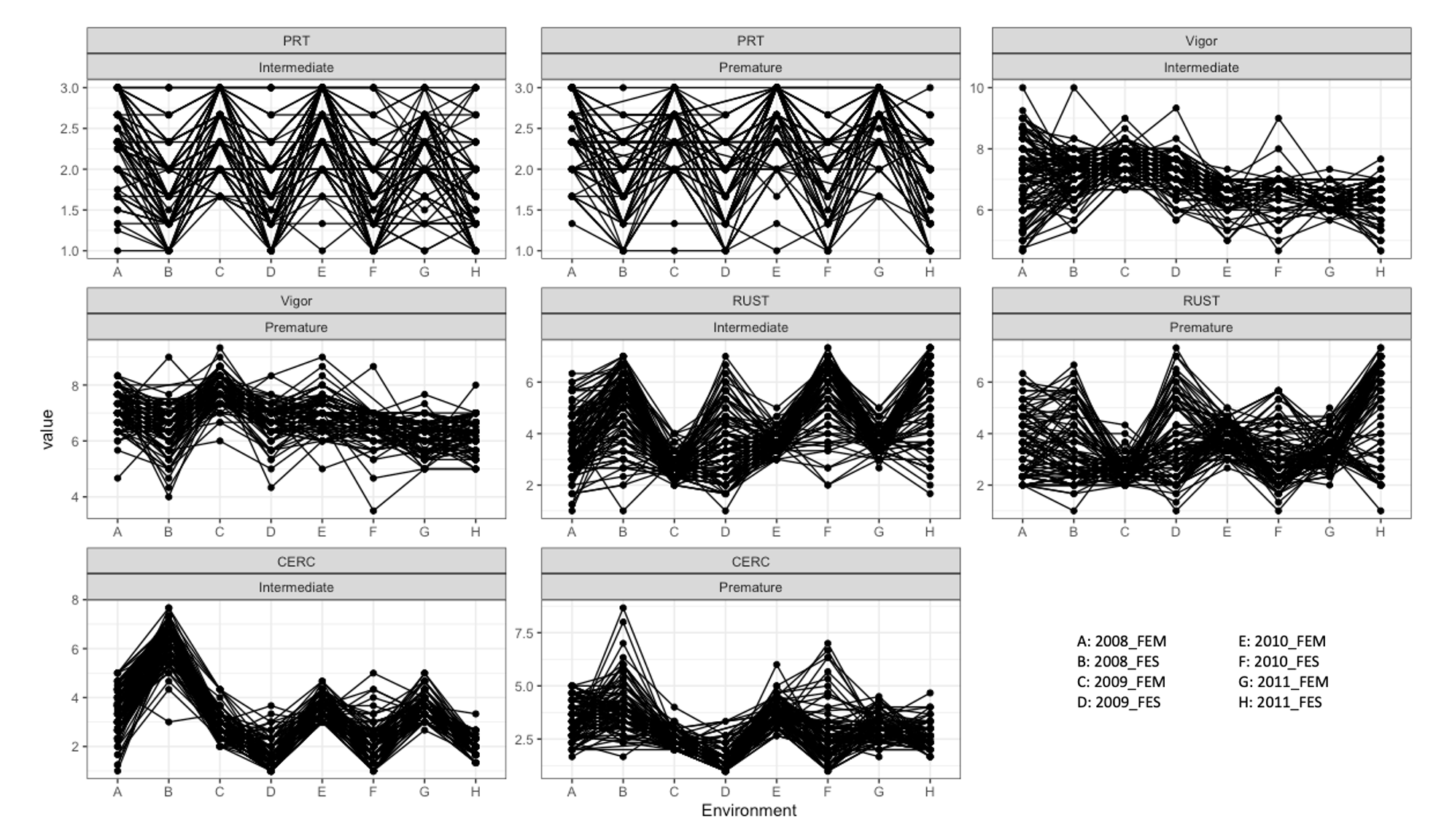


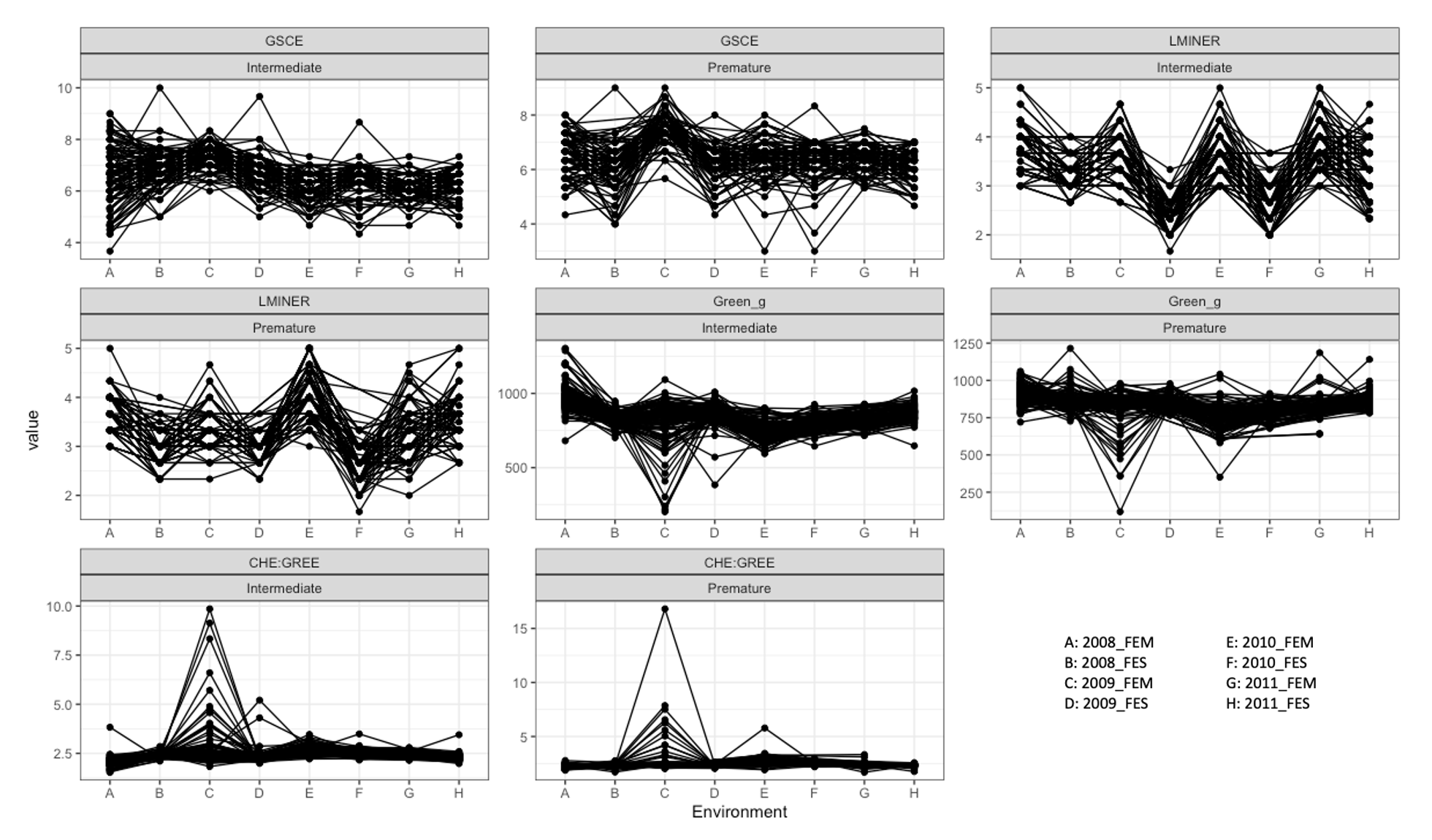


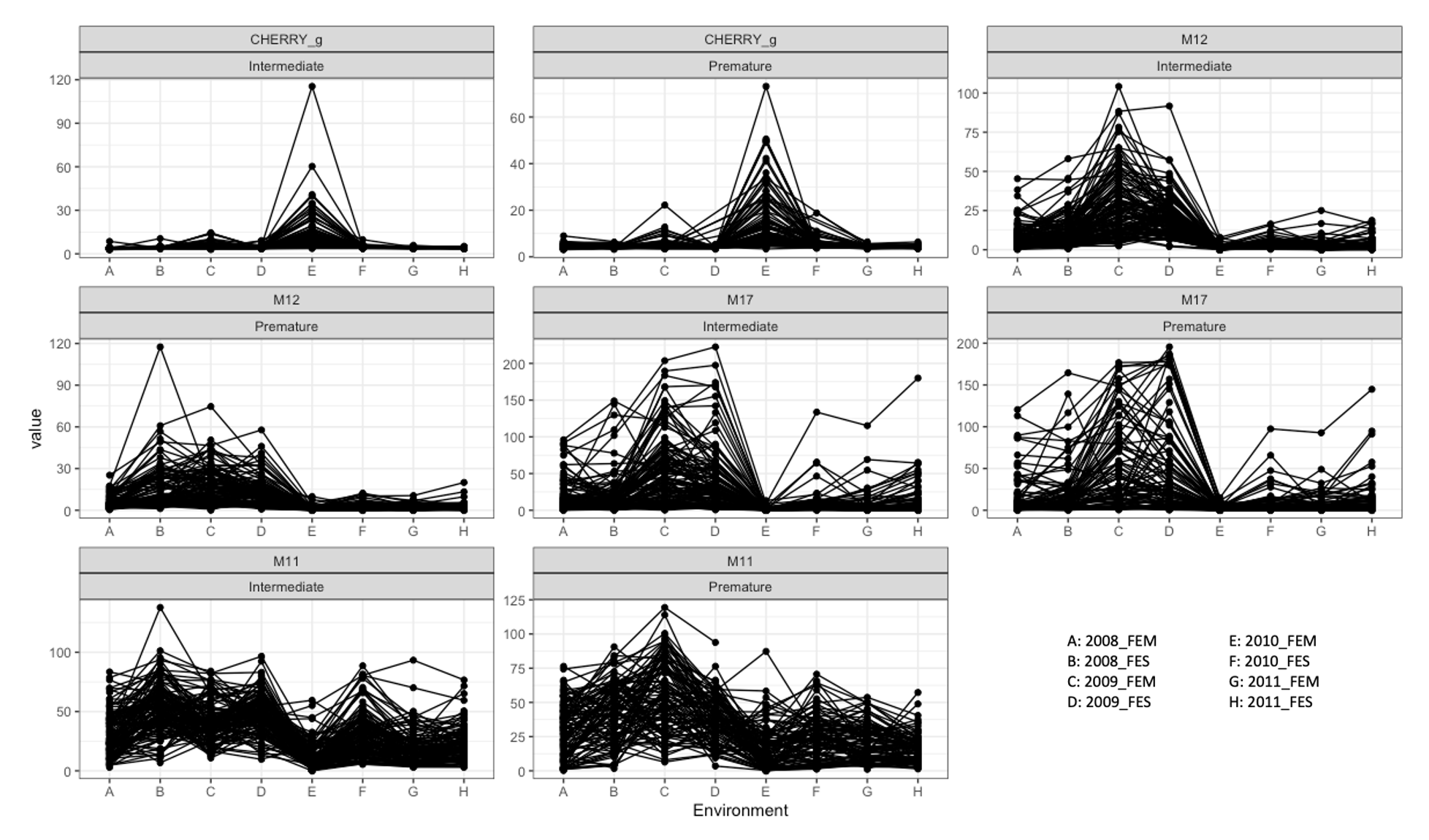


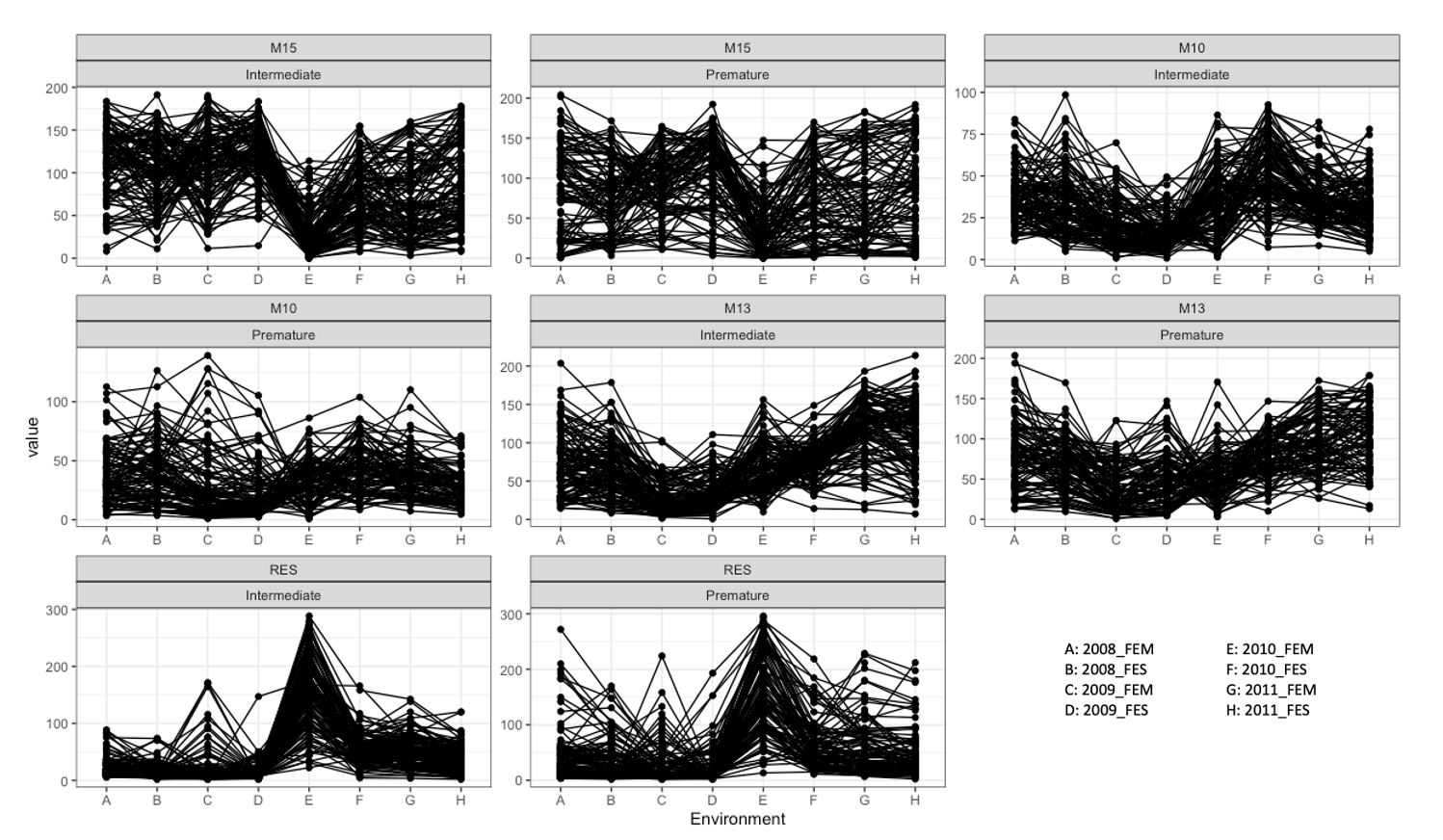


Figure S6. Graphical representation of genotype by environment interaction in two coffee populations.


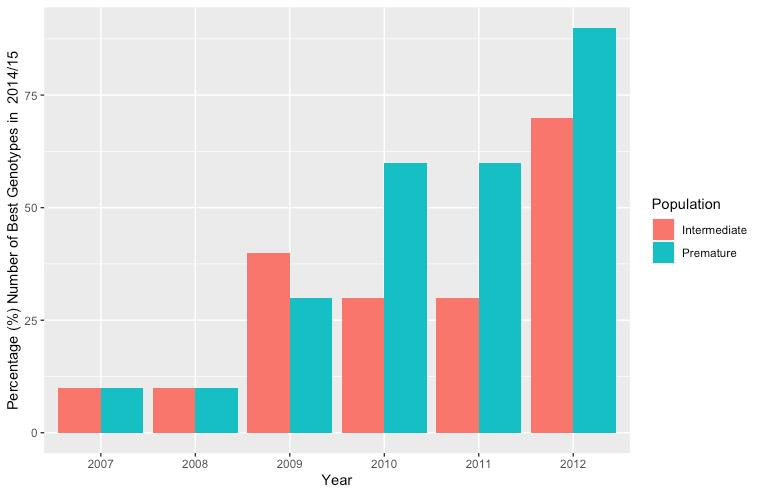


**Figure S7. Bar plot comparing the percentage top genotypes selected in each validation set based on their GEBVs to the top genotypes selected across years.**

Table S1. Summary of variance components (%) for selected coffee traits in premature and intermediate populations and the percentage of genotype by environment (GEI) variation explained by first two principal components (PC) in AMMI analysis

| Trait | Estimated variance-covariance | | | | % of GEI in | |
| --- | --- | --- | --- | --- | --- | --- |
|  | **Genotype** | **Environment** | **GEI** | **Residual** | **PC1** | **PC2** |
|  |  |  |  |  |  |  |
| Premature |  |  |  |  |  |  |
|  |  |  |  |  |  |  |
| GSCE | 4.80 | 93.39 | 1.21 | 0.60 | 28.4 | 27.7 |
| FL | 4.48 | 94.81 | 0.51 | 0.20 | 34.6 | 17.0 |
| VIGOR | 3.36 | 95.36 | 0.82 | 0.46 | 28.5 | 24.8 |
| UNIF | 0.83 | 98.65 | 0.29 | 0.23 | 25.3 | 22.3 |
| PRT | 5.56 | 93.62 | 0.43 | 0.38 | 58.9 | 14.7 |
| CERC | 1.74 | 97.21 | 0.78 | 0.28 | 42.5 | 17.4 |
| LMINER | 1.05 | 97.72 | 0.67 | 0.56 | 27.0 | 21.3 |
| RUST | 7.35 | 90.58 | 1.56 | 0.51 | 50.0 | 19.2 |
| GSIZ | 17.94 | 78.71 | 2.15 | 1.20 | 28.2 | 24.0 |
| CHERRY_g | 2.12 | 97.24 | 0.43 | 0.21 | 41.3 | 26.0 |
| CHE:GREE | 6.83 | 82.98 | 6.94 | 3.25 | 91.2 | 5.1 |
| GREEN_g | 3.33 | 93.73 | 1.84 | 1.10 | 45.9 | 21.9 |
| M10 | 25.18 | 68.31 | 5.17 | 1.34 | 46.6 | 15.2 |
| M11 | 4.45 | 93.52 | 1.32 | 0.70 | 41.4 | 17.7 |
| M12 | 4.23 | 94.07 | 1.10 | 0.59 | 53.7 | 27.9 |
| M13 | 6.53 | 91.69 | 1.29 | 0.49 | 30.3 | 20.3 |
| M15 | 19.28 | 77.41 | 2.48 | 0.83 | 44.8 | 17.0 |
| M17 | 11.38 | 86.65 | 1.64 | 0.33 | 63.7 | 23.2 |
| RES | 5.39 | 93.65 | 0.67 | 0.29 | 43.5 | 20.8 |
| YIELD | 2.38 | 96.91 | 0.54 | 0.18 | 30.5 | 26.3 |
|  |  |  |  |  |  |  |
| Intermediate |  |  |  |  |  |  |
|  |  |  |  |  |  |  |
| GSCE | 5.02 | 93.17 | 1.20 | 0.62 | 41.8 | 26.3 |
| FL | 2.67 | 96.79 | 0.37 | 0.17 | 36.1 | 20.6 |
| VIGOR | 3.81 | 94.53 | 1.13 | 0.53 | 50.4 | 22.4 |
| UNIF | 0.46 | 99.20 | 0.21 | 0.13 | 37.8 | 22.2 |
| PRT | 5.60 | 93.58 | 0.43 | 0.38 | 57.9 | 14.6 |
| CERC | 0.30 | 99.52 | 0.12 | 0.07 | 27.9 | 23.1 |
| LMINER | 0.87 | 98.47 | 0.36 | 0.30 | 25.6 | 18.0 |
| RUST | 1.84 | 97.50 | 0.47 | 0.18 | 34.5 | 21.6 |
| GSIZ | 4.63 | 94.81 | 0.37 | 0.19 | 37.6 | 20.1 |
| CHERRY_g | 0.97 | 98.53 | 0.32 | 0.17 | 39.7 | 32.3 |
| CHE:GREE | 2.84 | 93.10 | 2.93 | 1.13 | 87.1 | 6.2 |
| GREEN_g | 2.08 | 96.79 | 0.71 | 0.41 | 59.5 | 17.5 |
| M10 | 4.46 | 93.55 | 1.27 | 0.73 | 36.2 | 22.7 |
| M11 | 2.80 | 96.09 | 0.72 | 0.39 | 30.3 | 24.7 |
| M12 | 2.42 | 96.86 | 0.51 | 0.21 | 68.2 | 16.3 |
| M13 | 3.22 | 96.08 | 0.48 | 0.22 | 36.2 | 20.5 |
| M15 | 2.91 | 95.84 | 0.98 | 0.27 | 49.8 | 16.0 |
| M17 | 7.44 | 91.27 | 1.05 | 0.24 | 59.2 | 17.1 |
| RES | 0.88 | 98.68 | 0.29 | 0.15 | 57.8 | 18.8 |
| YIELD | 1.35 | 98.15 | 0.37 | 0.13 | 29.8 | 20.8 |
